# Supplementary material for: Rational design of microRNA-responsive switch for programmable translational control in mammalian cells
Source: Nat Commun. 2023 Nov 8;14:7193. doi: 10.1038/s41467-023-43065-w (PMC10632459; doi:10.1038/s41467-023-43065-w)
Supplement: Supplementary file 1 — Supplementary Information [file 41467_2023_43065_MOESM1_ESM.pdf]

Supplementary Information for

Rational design of microRNA-responsive switch for programmable translational control in mammalian cells

Hui Ning[1, 3], Gan Liu[2, 3], Lei Li[1], Qiang Liu[2], Huiya Huang[2], Zhen Xie[1,*]

1 MOE Key Laboratory of Bioinformatics and Bioinformatics Division, Center for Synthetic and System Biology, Department of Automation, Beijing National Research Center for Information Science and Technology, Tsinghua University, Beijing, 100084, China.

2 Syngentech Inc., Zhongguancun Life Science Park, Changping District, Beijing 102206, China.

3 These authors contributed equally: Hui Ning, Gan Liu

* Corresponding author: zhenxie@tsinghua.edu.cn (Z.X.)

Table of Contents

Supplementary Figure 1 to Supplementary Figure 10

Supplementary Fig. 1 Raw data of nIRES, dIRES and rIRES expression levels

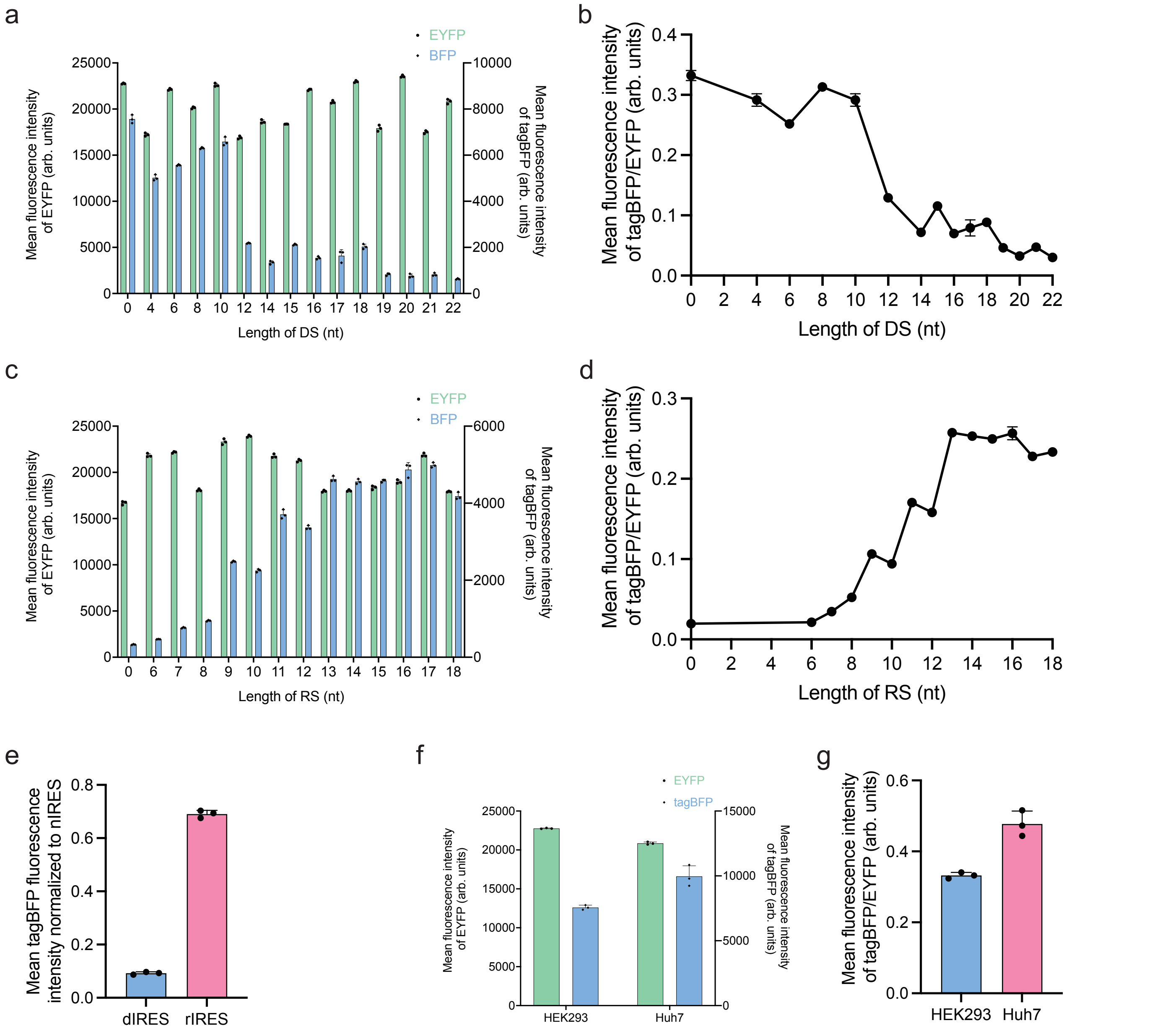

Supplementary Fig. 1 Raw data of nIRES, dIRES and rIRES expression levels.

(a-b) Raw data related to main Figure 1b. (a) Raw data of mean fluorescence intensity (MFI) of EYFP (internal control) and tagBFP (reporter of IRES translation activity). (b) Raw MFI ratio of tagBFP to EYFP of different length of DS. (c-d) Raw data related to main Figure 1d. (c) Raw data of MFI of EYFP (internal control) and tagBFP (reporter of IRES translation activity). (d) Raw MFI ratio of tagBFP to EYFP of different length of RS. (e) Normalized MFI of tagBFP of dIRES and rIRES in Huh7 cells. (f) Raw MFI data of nIRES in HEK293 and Huh7 cells. (g) Raw MFI ratio data of nIRES in HEK293 and Huh7 cells indicated the nIRES translation activity was slightly higher in Huh7 than in HEK293 with plasmid transfection. Data are presented as mean values with error bars representing standard deviation of three independent biological replicates (n=3 for each group). arb. units, arbitrary units.

Supplementary Fig. 2 Raw data and control experiments for miR-FF4 responsive MITAs

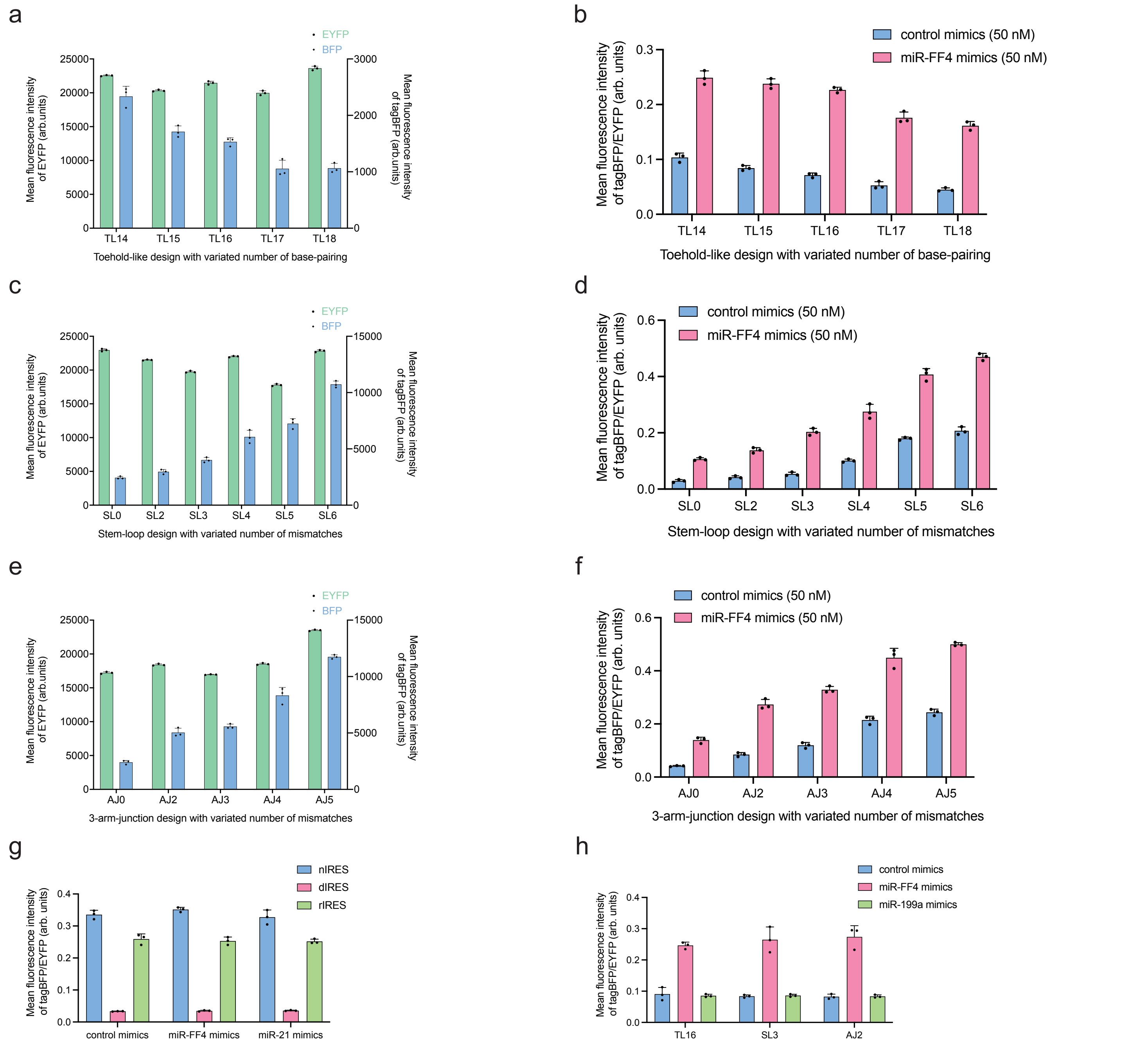

Supplementary Fig. 2 Raw data and control experiments for miR-FF4 responsive MITAs.

(a-b) Raw data related to main Figure 2d. (a) Raw data of MFI of EYFP (internal control) and tagBFP (reporter of IRES translation activity), related to different miR-FF4 responsive TL designed MITAs. (b) Raw MFI ratio of tagBFP to EYFP of different miR-FF4 responsive TL designed MITAs. (c-d) Raw data related to main Figure 2e. (c) Raw data of MFI of EYFP (internal control) and tagBFP (reporter of IRES translation activity), related to different miR-FF4 responsive SL designed MITAs. (d) Raw MFI ratio of tagBFP to EYFP of different miR-FF4 responsive SL designed MITAs. (e-f) Raw data related to main Figure 2f. (e) Raw data of MFI of EYFP (internal control) and tagBFP (reporter of IRES translation activity), related to different miR-FF4 responsive AJ designed MITAs. (f) Raw MFI ratio of tagBFP to EYFP of different miR-FF4 responsive TL designed MITAs. (g-h) Control experiments related to main Figure 2d-2f. (g) Raw MFI ratio data of nIRES, dIRES and rIRES with miRNA mimics co-transfection. (h) Raw MFI ratio data of miR-FF4 responsive TL16, SL3 and AJ2 MITAs with miRNA mimics co-transfection. Data are presented as mean values with error bars representing standard deviation of three independent biological replicates (n=3 for each group). arb. units, arbitrary units.

Supplementary Fig. 3 Raw data of miRNA activity in HEK293 and Huh7 and raw data of logic-gate experiments

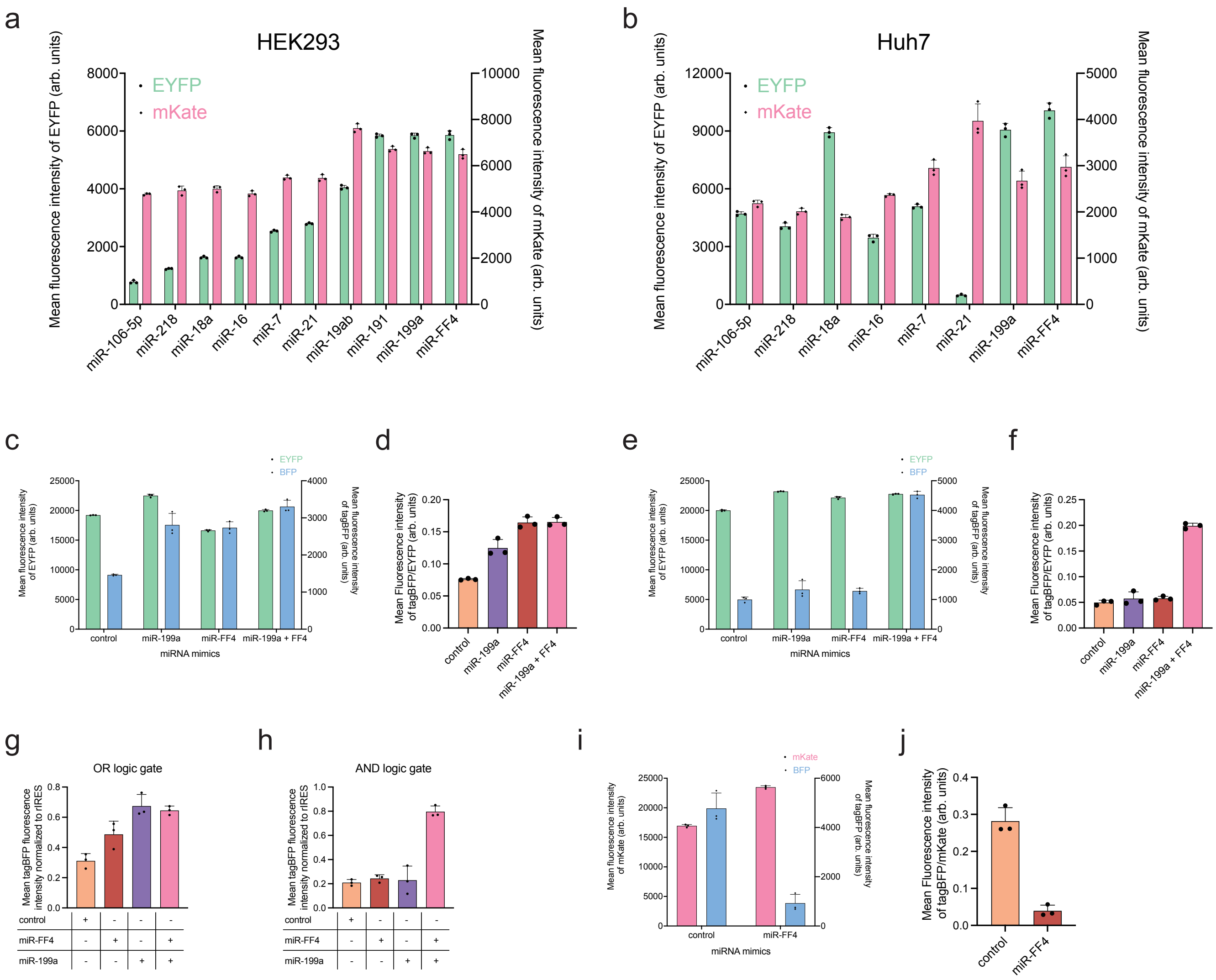

Supplementary Fig. 3 Raw data of miRNA activity in HEK293 and Huh7 and raw data of logic-gate experiments.

(a) Raw data of MFI of EYFP (reporter of miRNA repression activity) and mKate (co-transfected control fluorescence protein) in HEK293 cells. (b) Raw data of MFI of EYFP (reporter of miRNA repression activity) and mKate (co-transfected control fluorescence protein) in Huh7 cells. (c-d) Raw data related to main Figure 3b. (c) Raw data of MFI of EYFP (internal control) and tagBFP (reporter of IRES translation activity), related to OR logic gate design. (d) Raw MFI ratio of tagBFP to EYFP of OR logic gate design. (e-f) Raw data related to main Figure 3d. (e) Raw data of MFI of EYFP (internal control) and tagBFP (reporter of IRES translation activity), related to AND logic gate design. (f) Raw MFI ratio of tagBFP to EYFP of AND logic gate design. (g) Additional experiments of rearranging the order of miRBS in OR logic gate design. (h) Additional experiments of rearranging the order of miRBS in AND logic gate design. (i-j) Raw data related to main Figure 3f. (i) Raw data of MFI of mKate (co-transfected control fluorescence protein) and tagBFP (reporter of IRES translation activity), related to NOT logic gate (MITR) design. (j) Raw MFI ratio of tagBFP to mKate of the MITR design. Data are presented as mean values with error bars representing standard deviation of three independent biological replicates (n=3 for each group). arb. units, arbitrary units.

Supplementary Fig. 4 Raw data and control experiments for endogenous miRNA responsive MITAs and cell-type classifiers

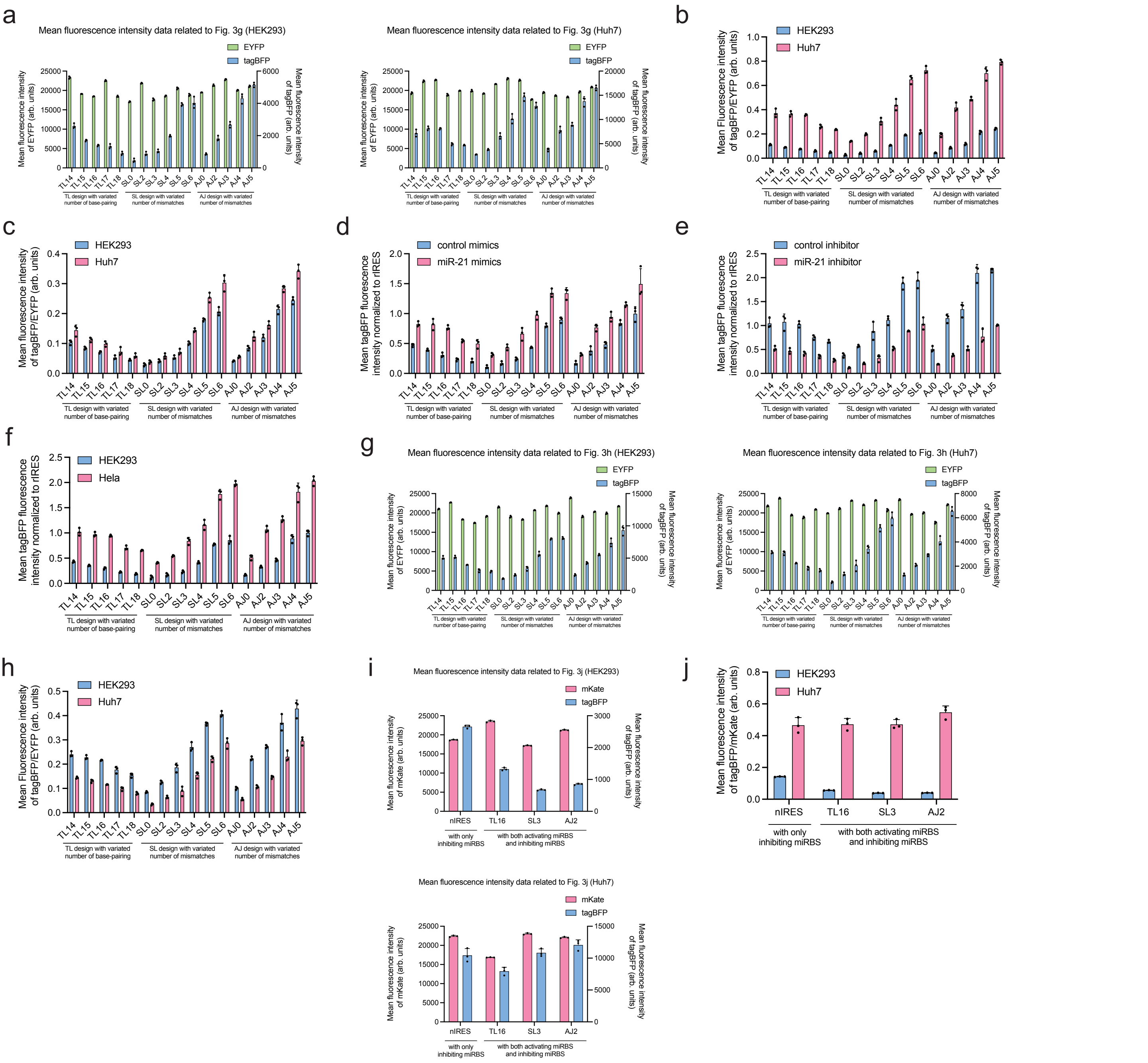

Supplementary Fig. 4 Raw data and control experiments for endogenous miRNA responsive MITAs and cell-type classifiers.

(a-b) Raw data related to main Figure 3g. (a) Raw data of MFI of EYFP (internal control) and tagBFP (reporter of IRES translation activity) in HEK293 (left panel) and Huh7 (right panel) cells, related to different miR-21 responsive MITAs. (b) Raw MFI ratio of tagBFP to EYFP of different miR-21 responsive MITAs in HEK293 and Huh7 cells. (c-e) Control experiments related to main Figure 3g. (c) Raw MFI ratio data of different miR-FF4 responsive MITAs co-transfected with control mimic (not miR-FF4) in HEK293 and Huh7 cells. (d) Normalized MFI of tagBFP of different miR-21 responsive MITAs co-transfected with control or miR-21 mimic in HEK293 cells. (e) Normalized MFI of tagBFP of different miR-21 responsive MITAs co-transfected with control or miR-21 inhibitor in Huh7 cells. (f) Normalized MFI of tagBFP of different miR-21 responsive MITAs in HEK293 and Hela cells. (g-h) Raw data related to main Figure 3h. (g) Raw data of MFI of EYFP (internal control) and tagBFP (reporter of IRES translation activity) in HEK293 (left panel) and Huh7 (right panel) cells, related to different miR-18a responsive MITAs. (h) Raw MFI ratio of tagBFP to EYFP of different miR-18a responsive MITAs in HEK293 and Huh7 cells. (i-j) Raw data related to main Figure 3j. (i) Raw data of MFI of mKate (co-transfected control fluorescence protein) and tagBFP (reporter of IRES translation activity) in HEK293 (up panel) and Huh7 (bottom panel) cells, related to nIRES-containing or MITA-containing cell-type classifiers. (j) Raw MFI ratio of tagBFP to mKate of nIRES-containing or MITA-containing cell-type classifiers in HEK293 and Huh7 cells. Data are presented as mean values with error bars representing standard deviation of three independent biological replicates (n=3 for each group). arb. units, arbitrary units.

Supplementary Fig. 5 Raw data of IRES, MITAs, and cell-type classifiers in circRNA

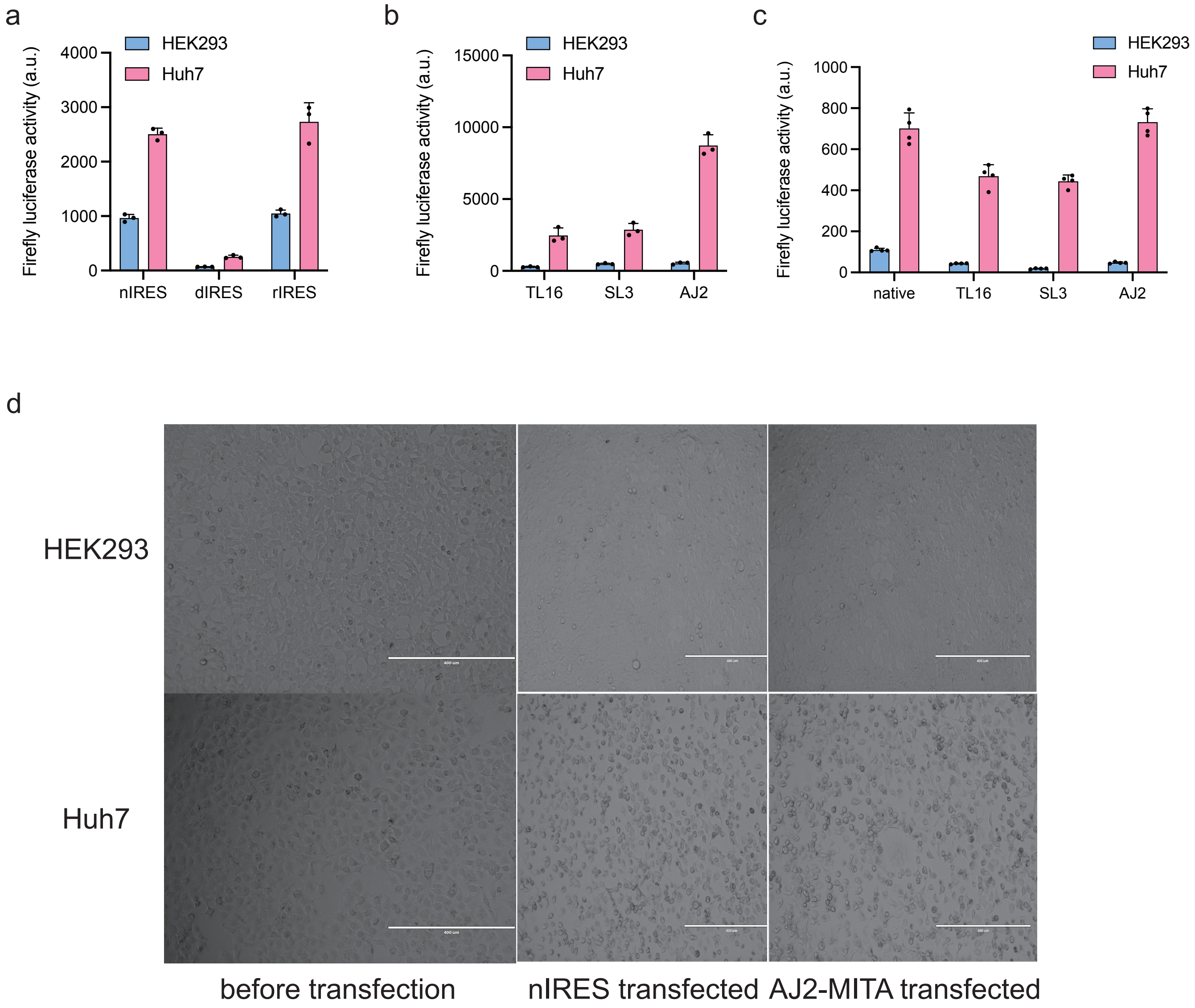

Supplementary Fig. 5 Raw data of IRES, MITAs, and cell-type classifiers in circRNA.

(a) Raw luminescence data of nIRES, dIRES and rIRES in circRNA in HEK293 and Huh7 cells, related to main Figure 4b. (b) Raw luminescence data of TL16, SL3 and AJ2 MITAs in circRNA in HEK293 and Huh7 cells, related to main Figure 4c. (c) Raw luminescence data of nIRES-containing or MITA-containing cell-type classifiers circRNAs in HEK293 and Huh7 cells, related to main Figure 4e. (d) Representative microscopy images of the transfection of GSDMD-encoding cell-type classifier circRNAs into HEK293 and Huh7 cells, related to main Figure 4i. Each experiment was repeated three times independently with similar results. Data are presented as mean values with error bars representing standard deviation of three independent biological replicates (n=3 for each group). a.u., arbitrary units.

Supplementary Fig. 6 Construction of mScarlet-HEK293 and EYFP-Huh7 stable cell lines

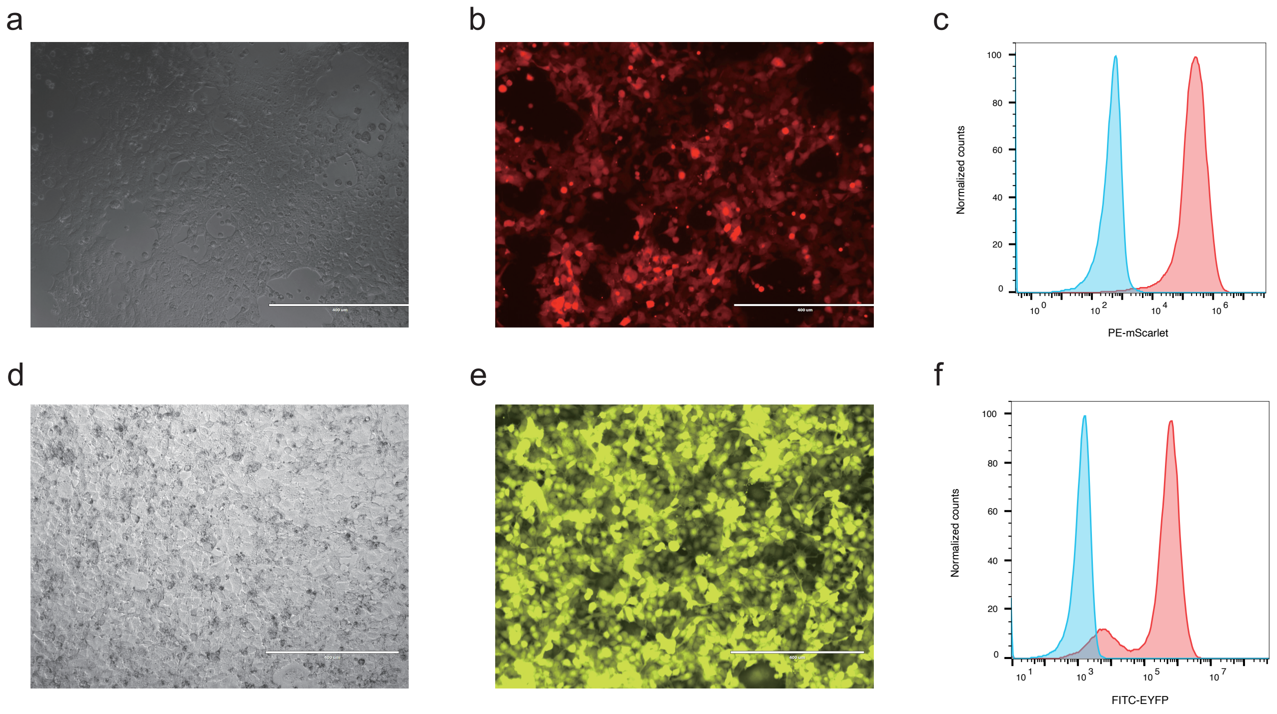

Supplementary Fig. 6 Construction of mScarlet-HEK293 and EYFP-Huh7 stable cell lines.

(a-c) Characterization of mScarlet-HEK293 stable cell lines through microscopy and flow cytometry. (a) Representative light microscopy images of mScarlet-HEK293 cells. (b) Representative fluorescence microscopy images of mScarlet-HEK293 cells. (c) Representative Histogram of flow cytometry characterizing the positive rate of mScarlet-HEK293 cells. The blue curve indicates the wild-type HEK293 cells and the red curve indicates the stably transfected mScarlet-HEK293 cells. (d-f) Characterization of EYFP-Huh7 stable cell lines through microscopy and flow cytometry. (d) Representative light microscopy images of EYFP-Huh7 cells. (e) Representative fluorescence microscopy images of EYFP-Huh7 cells. (f) Representative Histogram of flow cytometry characterizing the positive rate of EYFP-Huh7 cells. The blue curve indicates the wild-type Huh7 cells and the red curve indicates the stably transfected EYFP-Huh7 cells. Each experiment was repeated three times independently with similar results.

Supplementary Fig. 7 Cytotoxic cell-type classifiers selectively eliminate specific cell types

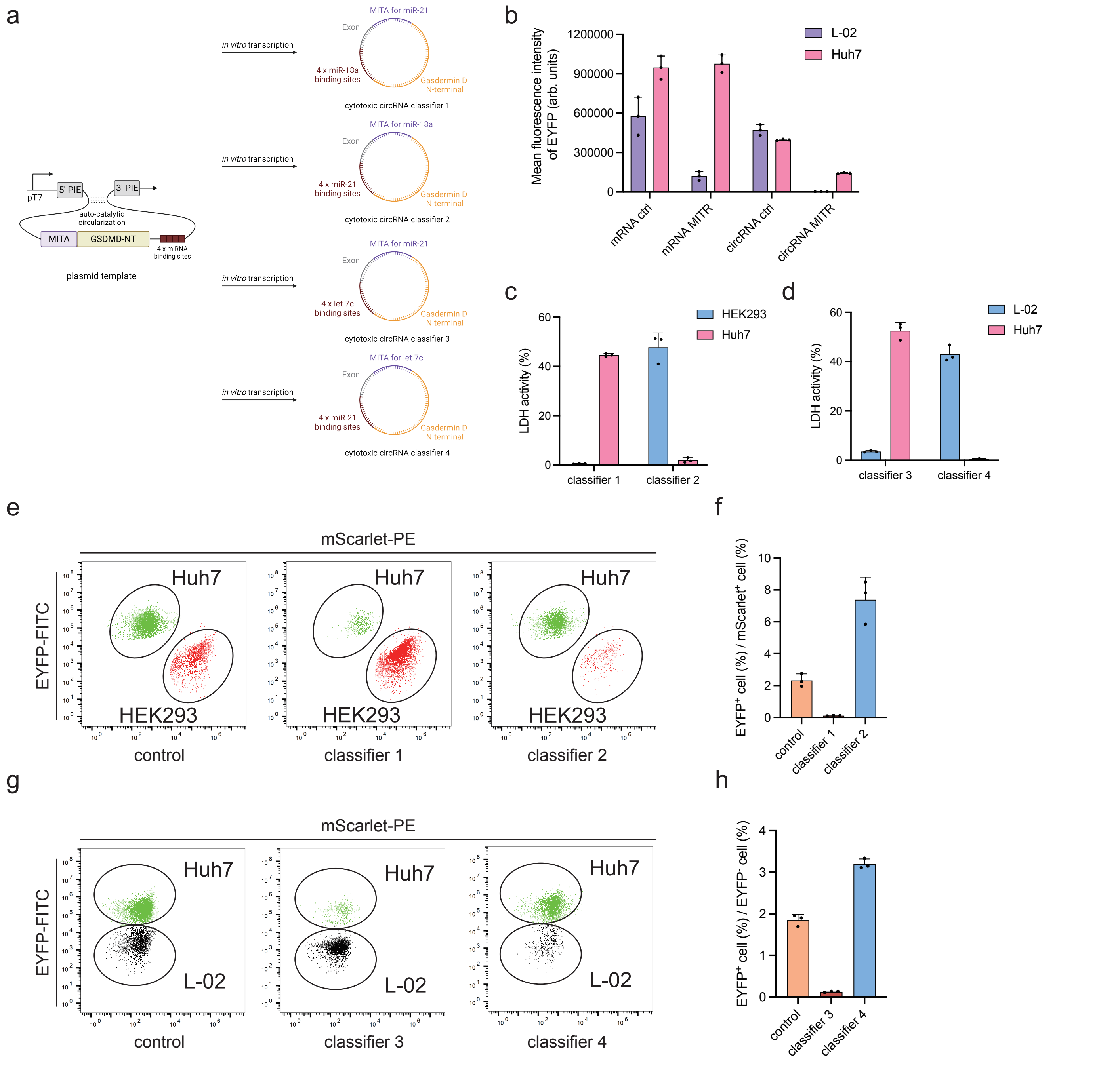

Supplementary Fig. 7 Cytotoxic cell-type classifiers selectively eliminate specific cell types.

(a) Schematic illustration of the plasmid constructs for in vitro transcription of cytotoxic cell-type classifier circRNAs. (b) Raw data of MFI of EGFP (reporter of let-7c miRNA repression activity) in Huh7 cells and L-02 cells. mRNA-ctrl: T7 promoter-EGFP-polyA; mRNA-MITR: T7 promoter-EGFP-4*let-7c binding sites-polyA; circRNA-ctrl: T7 promoter-5’PIE-IRES-EGFP-3’PIE; circRNA-MITR: T7 promoter-5’PIE-IRES-EGFP-4*let-7c binding sites-3’PIE. (c) Characterization of the cytotoxic GSDMD-encoding cell-type classifier 1 and classifier 2 circRNAs in Huh7 and HEK293 cells by LDH cytotoxic assays. (d) Characterization of the cytotoxic GSDMD-encoding cell-type classifier 3 and classifier 4 circRNAs in Huh7 and L-02 cells by LDH cytotoxic assays. (e) The scatter plots show the fractions of Huh7 (EYFP+) cells and HEK293 (mScarlet+) cells under the indicated conditions. (f) The bar chart shows the ratio of the surviving Huh7 (EYFP+) cell percentage to the surviving HEK293 (mScarlet+) cell percentage under the indicated conditions. (g) The scatter plots show the fractions of Huh7 (EYFP+) cells and L-02 (EYFP-) cells under the indicated conditions. (h) The bar chart shows the ratio of the surviving Huh7 (EYFP+) cell percentage to the surviving L-02 (EYFP-) cell percentage under the indicated conditions. Data are presented as mean values with error bars representing standard deviation of three independent biological replicates (n=3 for each group). Schematic illustration figures were created with BioRender.com with publication licenses. arb. units, arbitrary units.

Supplementary Fig. 8 Representative microscopy images of the transfection of GSDMD-encoding cell-type classifier circRNAs into HEK293, Huh7 and L-02 cells

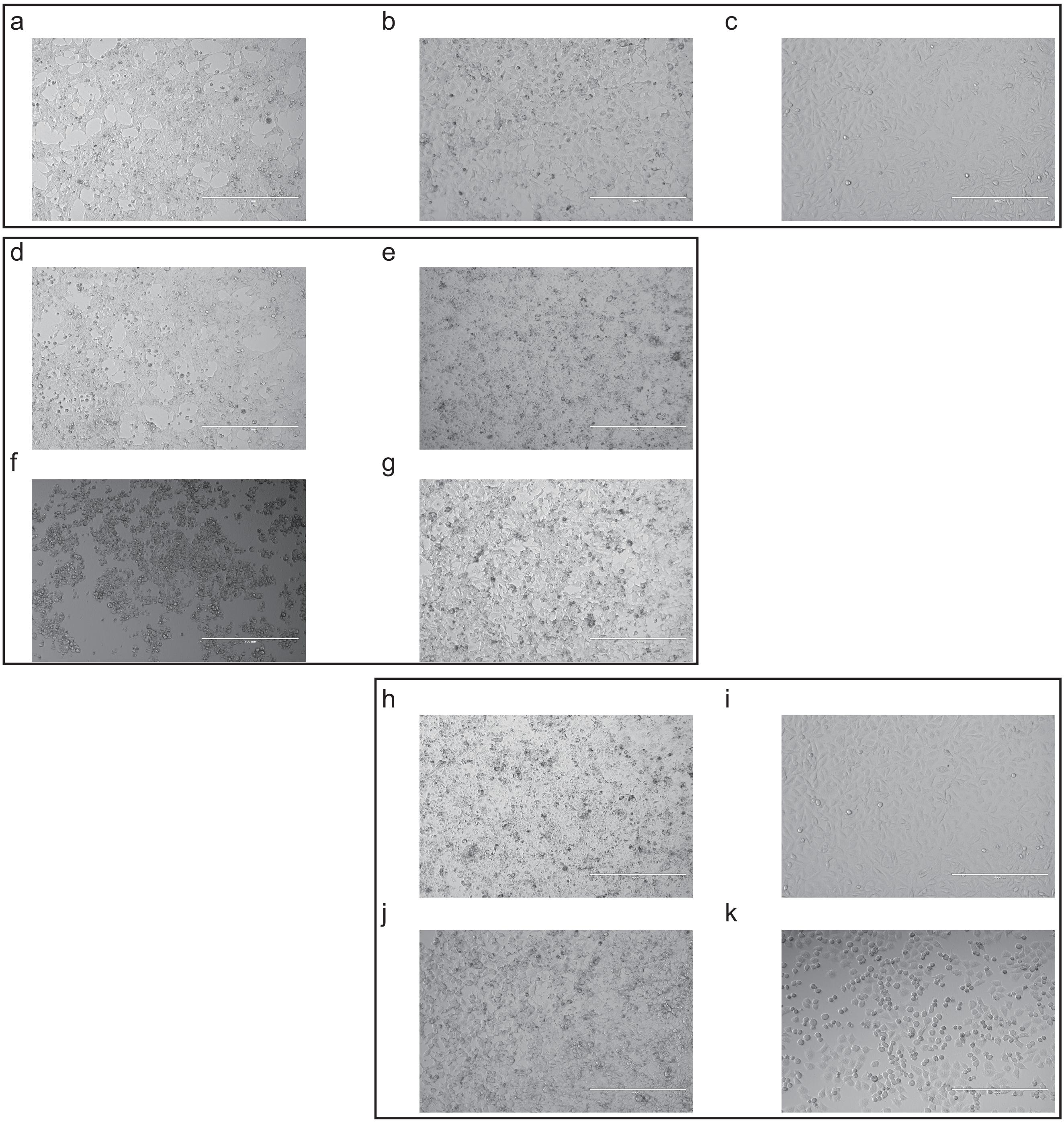

Supplementary Fig. 8 Representative microscopy images of the transfection of GSDMD-encoding cell-type classifier circRNAs into HEK293, Huh7 and L-02 cells.

(a) HEK293 cells transfected with empty LNPs. (b) Huh7 cells transfected with empty LNPs. (c) L-02 cells transfected with empty LNPs. (d) HEK293 cells transfected with LNP encapsulating classifier 1 circRNAs. (e) Huh7 cells transfected with LNP encapsulating classifier 1 circRNAs. (f) HEK293 cells transfected with LNP encapsulating classifier 2 circRNAs. (g) Huh7 cells transfected with LNP encapsulating classifier 2 circRNAs. (h) Huh7 cells transfected with LNP encapsulating classifier 3 circRNAs. (i) L-02 cells transfected with LNP encapsulating classifier 3 circRNAs. (j) Huh7 cells transfected with LNP encapsulating classifier 4 circRNAs. (k) L-02 cells transfected with LNP encapsulating classifier 4 circRNAs. Each experiment was repeated three times independently with similar results.

Supplementary Fig. 9 Raw data of IRES, MITA and MITR expression levels derived from CSFV IRES

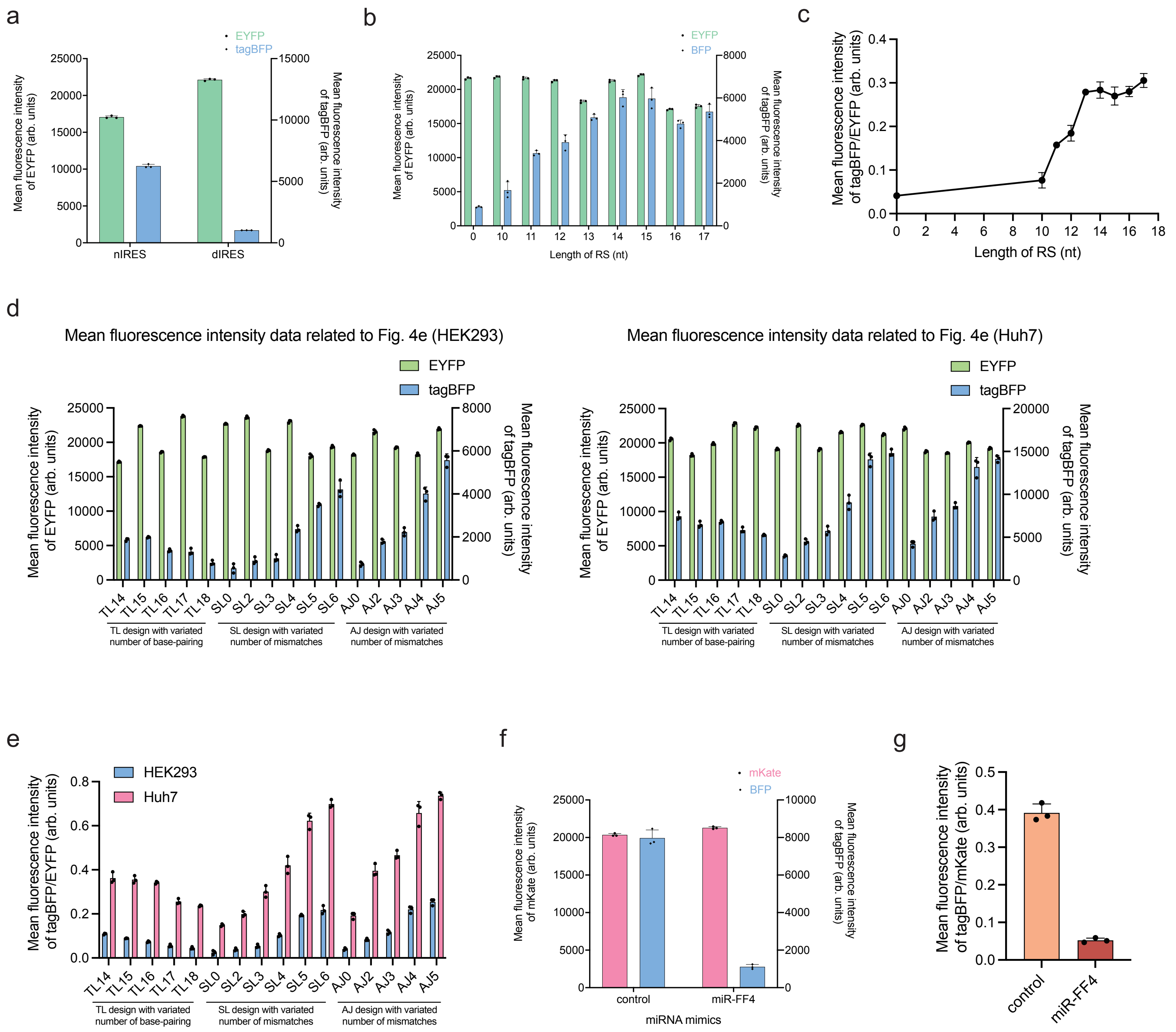

Supplementary Fig. 9 Raw data of IRES, MITA and MITR expression levels derived from CSFV IRES.

(a) Raw data of MFI of EYFP (internal control) and tagBFP (reporter of CSFV IRES translation activity) of nIRES and dIRES of CSFV IRES related to main Figure 5b. (b-c) Raw fluorescence data related to main Figure 5c-d. (b) Raw data of MFI of EYFP (internal control) and tagBFP (reporter of CSFV IRES translation activity). (c) Raw MFI ratio of tagBFP to EYFP of different length of RS of CSFV dIRES. (d-e) Raw data related to main Figure 5e. (d) Raw data of MFI of EYFP (internal control) and tagBFP (reporter of IRES translation activity) in HEK293 (left panel) and Huh7 (right panel) cells, related to different miR-21 responsive MITAs derived from CSFV IRES. (e) Raw MFI ratio of tagBFP to EYFP of different miR-21 responsive MITAs derived from CSFV IRES in HEK293 and Huh7 cells. (f-g) Raw data related to main Figure 5g. (f) Raw data of MFI of mKate (co-transfected control fluorescence protein) and tagBFP (reporter of CSFV IRES translation activity), related to NOT logic gate (MITR) derived from CSFV IRES. (g) Raw MFI ratio of tagBFP to mKate of the MITR design derived from CSFV IRES. Data are presented as mean values with error bars representing standard deviation of three independent biological replicates (n=3 for each group). arb. units, arbitrary units.

Supplementary Fig. 10 Demonstration of flow cytometry gating strategy

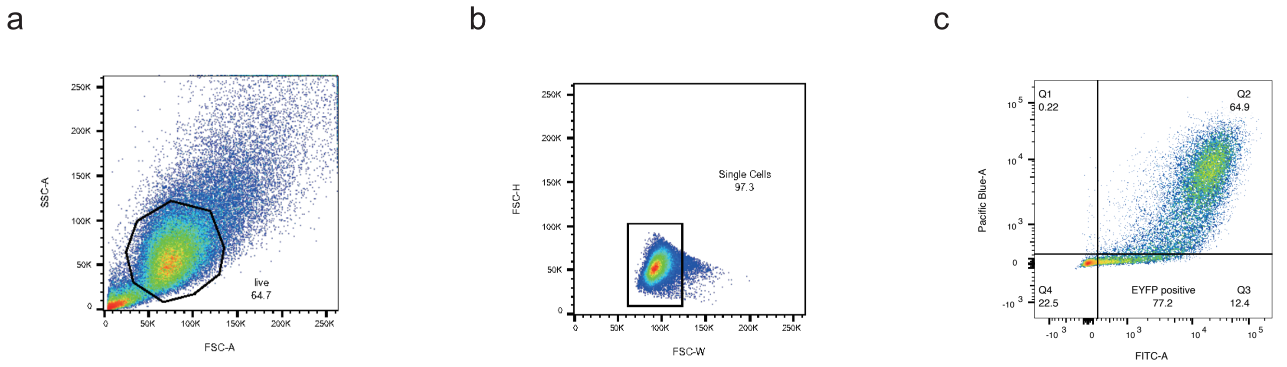

Supplementary Fig. 10 Demonstration of flow cytometry gating strategy.

(a) Gating for living cells. (b) Gating for single cells. (c) Gating for reporter-positive cells. Each experiment was repeated three times independently with similar results.
