# Supplementary material for: Rational design of microRNA-responsive switch for programmable translational control in mammalian cells
Source: Nat Commun. 2023 Nov 8;14:7193. doi: 10.1038/s41467-023-43065-w (PMC10632459; doi:10.1038/s41467-023-43065-w)
Supplement: Supplementary file 3 — Reporting Summary [file 41467_2023_43065_MOESM3_ESM.pdf]

nature portfolio

Corresponding author(s): Zhen Xie

Last updated by author(s): Oct 19, 2023

# Reporting Summary

Nature Portfolio wishes to improve the reproducibility of the work that we publish. This form provides structure for consistency and transparency in reporting. For further information on Nature Portfolio policies, see our Editorial Policies and the Editorial Policy Checklist.

## Statistics

For all statistical analyses, confirm that the following items are present in the figure legend, table legend, main text, or Methods section.

| n/a | Confirmed | |
|---|---|---|
| ☐ | ☒ | The exact sample size ($n$) for each experimental group/condition, given as a discrete number and unit of measurement |
| ☐ | ☒ | A statement on whether measurements were taken from distinct samples or whether the same sample was measured repeatedly |
| ☐ | ☒ | The statistical test(s) used AND whether they are one- or two-sided<br>*Only common tests should be described solely by name; describe more complex techniques in the Methods section.* |
| ☒ | ☐ | A description of all covariates tested |
| ☐ | ☒ | A description of any assumptions or corrections, such as tests of normality and adjustment for multiple comparisons |
| ☐ | ☒ | A full description of the statistical parameters including central tendency (e.g. means) or other basic estimates (e.g. regression coefficient) AND variation (e.g. standard deviation) or associated estimates of uncertainty (e.g. confidence intervals) |
| ☐ | ☒ | For null hypothesis testing, the test statistic (e.g. $F$, $t$, $r$) with confidence intervals, effect sizes, degrees of freedom and $P$ value noted<br>*Give P values as exact values whenever suitable.* |
| ☒ | ☐ | For Bayesian analysis, information on the choice of priors and Markov chain Monte Carlo settings |
| ☒ | ☐ | For hierarchical and complex designs, identification of the appropriate level for tests and full reporting of outcomes |
| ☒ | ☐ | Estimates of effect sizes (e.g. Cohen's $d$, Pearson's $r$), indicating how they were calculated |

*Our web collection on statistics for biologists contains articles on many of the points above.*

## Software and code

Policy information about availability of computer code

| | |
|---|---|
| Data collection | Flow cytometry data was collected using BD FASCDiva Software(8.0.1) |
| Data analysis | FlowJo v10.8.1 (FlowJo, LLC)was used for flow cytometry data analysis.<br>Prism 9 (Version 9.4.1) was used for data analysis, including calculation of the means, standard deviations and P-values of statistical test.<br>Geneious Prime (Version 2022.0.1) was used for gene sequence analysis and primer design. |

For manuscripts utilizing custom algorithms or software that are central to the research but not yet described in published literature, software must be made available to editors and reviewers. We strongly encourage code deposition in a community repository (e.g. GitHub). See the Nature Portfolio guidelines for submitting code & software for further information.

## Data

Policy information about availability of data

All manuscripts must include a data availability statement. This statement should provide the following information, where applicable:

- Accession codes, unique identifiers, or web links for publicly available datasets
- A description of any restrictions on data availability
- For clinical datasets or third party data, please ensure that the statement adheres to our policy

All data supporting the findings of this study are available within the manuscript and its Supplementary Information and its Supplementary Data files. The sequence information has been provided within the Supplementary Data file. The raw data generated in this study have been provided within the Source Data file. The

annotated sequences have been deposited at Benchling website under the link https://benchling.com/hui_ning/f_/K81GiYaI-rational-design-of-microrna-responsive-switch-for-programmable-translational-control-in-mammalian-cells/. Source data are provided with this paper.

## Research involving human participants, their data, or biological material

Policy information about studies with human participants or human data. See also policy information about sex, gender (identity/presentation), and sexual orientation and race, ethnicity and racism.

| | |
|---|---|
| Reporting on sex and gender | N/A |
| Reporting on race, ethnicity, or other socially relevant groupings | N/A |
| Population characteristics | N/A |
| Recruitment | N/A |
| Ethics oversight | N/A |

Note that full information on the approval of the study protocol must also be provided in the manuscript.

# Field-specific reporting

Please select the one below that is the best fit for your research. If you are not sure, read the appropriate sections before making your selection.

☒ Life sciences ☐ Behavioural & social sciences ☐ Ecological, evolutionary & environmental sciences

For a reference copy of the document with all sections, see nature.com/documents/nr-reporting-summary-flat.pdf

# Life sciences study design

All studies must disclose on these points even when the disclosure is negative.

| | |
|---|---|
| Sample size | No special sample size calculations were performed. By convention, experiments were performed in independent biological triplicate (n=3). |
| Data exclusions | No data was excluded from analysis. |
| Replication | The activity of the IRES reporter was measured in three biological replicates. All attempts at replication were successful. |
| Randomization | Randomization is not relevant to our study, as all experiments were done in cell culture. Samples were separated based on transfection conditions. |
| Blinding | Blinding is not applicable in our study, as all experiments were done in cell culture. |

# Reporting for specific materials, systems and methods

We require information from authors about some types of materials, experimental systems and methods used in many studies. Here, indicate whether each material, system or method listed is relevant to your study. If you are not sure if a list item applies to your research, read the appropriate section before selecting a response.

## Materials & experimental systems

| n/a | Involved in the study | |
|---|---|---|
| ☒ | ☐ | Antibodies |
| ☐ | ☒ | Eukaryotic cell lines |
| ☒ | ☐ | Palaeontology and archaeology |
| ☒ | ☐ | Animals and other organisms |
| ☒ | ☐ | Clinical data |
| ☒ | ☐ | Dual use research of concern |
| ☒ | ☐ | Plants |

## Methods

| n/a | Involved in the study | |
|---|---|---|
| ☒ | ☐ | ChIP-seq |
| ☐ | ☒ | Flow cytometry |
| ☒ | ☐ | MRI-based neuroimaging |

# Eukaryotic cell lines

Policy information about cell lines and Sex and Gender in Research

| | |
|---|---|
| Cell line source(s) | HEK293 (293FT, Life Technologies); Huh7 (BeNa culture collection Co., Ltd); Hela (ATCC); L-02 (ATCC) |
| Authentication | HEK293 (293FT) cell line was authenticated upon purchased from Life Technologies (STR profile validated); Huh7 cell line was authenticated upon purchased from BeNa culture coleection Co., Ltd (STR profile validated). Hela and L-02 cell lines were authenticated upon purchased from ATCC (STR profile validated). |
| Mycoplasma contamination | Cell lines were tested negative for mycoplasma contamination at the time of purchase. The cell line did not exhibit any abnormal features during subsequent culture. |
| Commonly misidentified lines (See ICLAC register) | No commonly misidentified cell lines were used. |

# Flow Cytometry

## Plots

Confirm that:

☒ The axis labels state the marker and fluorochrome used (e.g. CD4-FITC).

☒ The axis scales are clearly visible. Include numbers along axes only for bottom left plot of group (a 'group' is an analysis of identical markers).

☒ All plots are contour plots with outliers or pseudocolor plots.

☒ A numerical value for number of cells or percentage (with statistics) is provided.

## Methodology

| | |
|---|---|
| Sample preparation | Cells were trypsinized 3 days after transfection and centrifuged at 300 g for 5 min at room temperature. The supernatant was removed, and the cells were resuspended in phosphate-buffered saline (PBS) that did not contain calcium or magnesium. |
| Instrument | LSRFortessa (BD Biosciences) |
| Software | Collect: BD FASCDiva Software (8.0.1)<br>Analyze: FlowJo (10.8.1) |
| Cell population abundance | EYFP-positive or tagBFP-positive in transfected cells were typically over 60% of the population. |
| Gating strategy | Negative control and fluorophore-positive cells were used to establish gates for each cell types. A figure exemplifying the gating strategy is provided in the Supplementary Information. |

☒ Tick this box to confirm that a figure exemplifying the gating strategy is provided in the Supplementary Information.
