# Supplementary material for: Rational design of microRNA-responsive switch for programmable translational control in mammalian cells
Source: Nat Commun. 2023 Nov 8;14:7193. doi: 10.1038/s41467-023-43065-w (PMC10632459; doi:10.1038/s41467-023-43065-w)
Supplement: Supplementary file 4 — Description of Additional Supplementary Files [file 41467_2023_43065_MOESM4_ESM.pdf]

Description of Additional Supplementary Files

File name:

Supplementary Data 1

Description:

This Excel file shows the sequences of plasmids, miRNA mimics and inhibitors used in the study.

See the following link at the benchling website for detailed sequence annotations:

https://benchling.com/hui_ning/f_/K81GiYaI-rational-design-of-microrna-responsive-switch-for-programmable-translational-control-in-mammalian-cells/
